# Efficacy of Fangfeng Tongsheng Granule Combined with Levocetirizine in the Treatment of Chronic Urticaria and Its Effect on Serum Complement, IL-4, IgE, and IFN-*γ* Levels in Patients

**DOI:** 10.1155/2022/4012416

**Published:** 2022-09-30

**Authors:** Duanni Xu, Zhenjie Li, Yinan Wang

**Affiliations:** ^1^Department of Outpatient, Guangzhou Institute of Dermatology, Guangzhou, Guangdong 510000, China; ^2^Department of Dermatology, Hospital of Traditional Chinese Medicine of Yantai City, Yantai, Shandong 264000, China

## Abstract

**Objectives:**

To investigate the efficacy of Fangfeng Tongsheng granule combined with levocetirizine in the treatment of chronic urticaria and its effect on serum complement, interleukin (IL)-4, immunoglobulin E (IgE), and interferon-*γ* (IFN-*γ*) levels in patients.

**Methods:**

A total of 98 patients with chronic urticaria who were admitted to our hospital from July 2021 to March 2022 were selected and divided into random odd-even numbers. The odd numbers were included in the observation group, with a total of 49 cases, and they were treated with Fangfeng Tongsheng granule combined with levocetirizine; the even numbers were included in the control group, with a total of 49 cases and were treated with levocetirizine alone. The two groups of patients were treated continuously for 4 weeks, and the clinical efficacy of the two groups was observed. Before treatment, 2 weeks and 4 weeks after treatment, evaluate the clinical symptom scores of patients such as itching, flushing, wheal, edema, observe the improvement of clinical symptoms of patients, and the changes in Dermatology Life Quality Index (DLQI). Serum complement C3, C4, T lymphocyte subsets CD_3_^+^, CD_4_^+^, CD_8_^+^ levels and CD_4_^+^/CD_8_^+^ ratio, IL-4, IgE, and IFN-*γ* levels and the occurrence of adverse reactions in the two groups were calculated and observed. All patients were followed up for 2 months after treatment to observe the recurrence of patients.

**Results:**

The scores of clinical symptoms such as wheal, itching, flushing, edema, and attack frequency in the observation group at each time point after treatment were lower than those in the control group (*F* times were 725.365, 851.521, 936.411, 3943.136, and 2226.147, all *P* < 0.05 (*F* between-group were 40.642, 102.124, 188.523, 259.291, and 23.92, *P* < 0.05); the total effective rate of the observation group was 93.88% (46/49), which was significantly higher than that of the control group, 73.47% (36/49) (*χ*^2^ = 7.470, *P*=0.006). The DLQI scores of the observation group at each time point after treatment were lower than those of the control group (*F* time was 282.214, *P* < 0.05; *F* between-group was 6.546, *P* < 0.05). There was no significant difference in serum C4 levels between the two groups at each time point (*F* time was 1.225, *P* > 0.05; *F* between-group was 0.408, *P* > 0.05); serum complement C3, CD_3_^+^, and CD_4_^+^/the ratio of CD_8_^+^ and IFN-*γ* were higher than those in the control group (*F* time was 407.352, 107.823, 32.941, and 2354.147, *P* < 0.05; *F* between-group was 40.941, 24.710, 54.982, and 264.921, *P* < 0.05); the observation group at each time after treatment the levels of IgE and IL-4 were lower than those of the control group (*F* time were 373.124 and 395.612, *P* < 0.05; *F* between-group were 21.802 and 62.591, *P* < 0.05). The incidence of adverse reactions in the observation group was 12.24% (6/49) compared with 10.20% (5/49) in the control group, which had no significant difference (*χ*^2^ = 0.102, *P*=0.749). Both groups were followed up for 2 months after treatment. The recurrence rate in the observation group was 12.24% (6/49), which was lower than that in the control group, which was 32.65% (16/49) (*χ*^2^ = 5.861, *P*=0.015).

**Conclusion:**

The application of Fangfeng Tongsheng granules combined with levocetirizine in patients with chronic urticaria can effectively improve the clinical symptoms of patients, improve clinical efficacy, reduce the impact of the disease on life, improve the immune status of patients, and reduce the risk of recurrence.

## 1. Introduction

Urticaria is a common allergic skin disease, of which chronic urticaria is the most common, which can recur frequently and last for months or years. Chronic urticaria is characterized by irregular wheals in the skin, often accompanied by erythema, pruritus, or local edema response, with the course of disease often exceeding six weeks [1]. Chronic urticaria is stubborn and complicated in etiology. It often recurs after drug withdrawal, which brings great trouble to the daily life of patients [2]. Histamine is a commonly used drug in the clinical treatment, of which levocetirizine is the most common, and its clinical efficacy has been recognized, but there are still some patients with poor drug response and unsatisfactory clinical effect [3–6]. Traditional Chinese medicine (TCM) classifies chronic urticaria into the category of “addiction rash,” and believes that it is caused by the invasion of “wind pathogens” and the insufficiency of external defenses. If the Qi is not dispersing, the disease occurs on the surface, and the treatment should be based on expelling wind and promoting blood circulation, strengthening the surface and benefiting Qi [7, 8]. Fangfeng Tongsheng granule is modified on Fangfeng Tongsheng powder created by Liu Wansu, one of the four famous artists in the Jin and Yuan Dynasties, and it has the effects of relieving exterior syndrome and internal invasion, sweating to exterior syndrome, dispelling wind, and reducing fever. Therefore, in this study, Fangfeng Tongsheng granule combined with levocetirizine was applied in the treatment of chronic urticaria, and its clinical efficacy and effects on the immune system and inflammatory response were observed, which was a clinical supplementary evidence-based basis.

## 2. Materials and Methods

### 2.1. General Information

A total of 98 patients with chronic urticaria who were admitted to our hospital from July 2021 to March 2022 were selected. The inclusion criteria include the following: ① patients who meet the clinical diagnostic criteria of chronic urticaria (urticaria occurs repeatedly for more than 6 weeks and at least twice a week, which is defined as chronic urticaria) [9]; ② patients who are in line with TCM “addiction rash” syndrome of gastrointestinal damp-heat [10], the main syndrome is wheal and flushing on the skin; the secondary symptoms are abdominal pain, chest tightness, loss of appetite, and anorexia; tongue and pulse symptoms are red tongue with yellow and greasy coating and slippery pulse; ③ the patients and their families understood the research content and gave written consent. The exclusion criteria include the following: ① patients who have taken immunosuppressive drugs or had related treatment in the past 3 months; ② patients with immune or blood system dysfunction; ③ women who are pregnant or breastfeeding; ④ patients with induced urticaria; ⑤ patients with severe organic impairment or malignant tumor; and ⑥ patients who are allergic to the drugs used in this study.

We grouped the patients by random parity number. Odd numbers were included in the observation group (*n* = 49); even numbers were included in the control group (*n* = 49). In the observation group, there were 24 males and 25 females; the age ranged from 20 to 42 years, with an average of (30.96 ± 5.08) years; the course of disease was 0.5 to 6 years, with an average of (3.21 ± 1.24) years. In the control group, there were 26 males and 23 females; the age ranged from 19 to 42 years, with an average of (30.42 ± 5.29) years; the course of disease was 0.5 to 6 years, with an average of (3.26 ± 1.14) years. There were no significant differences in the baseline data such as gender, age, and the course of disease between the two groups (sex: *χ*^2^ = 0.163, *P*=0.686; age: *t* = 0.515, *P*=0.607; the course of disease: *t* = 0.208, *P*=0.836).

### 2.2. Treatment Methods

Levocetirizine alone treatment (control group): levocetirizine hydrochloride tablets (Switzerland: UCB Farchim SA, registration number H20150522, 5 mg/tablet) were given orally, 5 mg/time, 1 time/day. Fangfeng Tongsheng granule combined with levocetirizine treatment (observation group): Fangfeng Tongsheng granule (Shandong Runzhong Pharmaceutical Co., Ltd., Z20174069, 3 g/bag) brewed with warm water, 1 bag/time, 2 times/day. The administration method of levocetirizine was the same as that of the control group. The patients in both the groups were treated continuously for 4 weeks.

### 2.3. Observation Indicators

#### 2.3.1. Clinical Symptom Score

Before treatment, 2 weeks and 4 weeks after treatment, the scores of clinical symptoms such as wheal, itching, flushing, edema, and attack frequency were evaluated in the two groups [11]. Each symptom was scored on a scale of 0 to 3 according to the severity. A lower score indicated milder symptoms.

#### 2.3.2. Clinical Efficacy

The results after 4 weeks of treatment were used as the criterion for the clinical efficacy evaluation [12]. Clinically cured: after treatment, the patient's symptom score was decreased by 95% or more as compared with that before treatment, and his wheal, flushing, and itching completely disappeared. Excellent: after treatment, the patient's symptom score decreased by 70%–94% as compared with that before treatment, his wheal mass dissipated by more than 70%, and his itching sensation was significantly reduced. Effective: after treatment, the patient's symptom score decreased by 30%–69% as compared with that before treatment, his wheal mass dissipated by more than 30%, and his itching sensation was relieved. Ineffective: patients who do not meet the above conditions are regarded as ineffective in treatment.

#### 2.3.3. Quality of Life

Before treatment, 2 weeks and 4 weeks after treatment, the Dermatology Life Quality Index (DLQI) [13] was used to observe the improvement of the degree of disease-related life impact of patients. The DLQI covers 10 aspects including psychology, physiology, entertainment, work, life, daily activities, and sports, with a total score of 0 to 30. The higher score means greater the impact of the disease on life.

#### 2.3.4. Serological Indicators

Before treatment, 2 weeks and 4 weeks after treatment, fasting venous blood was drawn from patients to detect peripheral blood serum complement C3, C4, T lymphocyte subsets CD_3_^+^, CD_4_^+^, CD_8_^+^ levels and calculated CD_4_^+^/CD_8_^+^ ratio, IL-4, IgE, and IFN-*γ* levels. Automatic biochemical analyzer (Roche cobas-c702) detected serum complement C3 and C4 levels, flow cytometer (Beckman CytoFLEX) detected CD_3_^+^, CD_4_^+^, and CD_8_^+^ levels; the levels of IL-4, IgE, and IFN-*γ* were detected by ELISA (kit: Shanghai Enzyme Research Biotechnology Co., Ltd.).

#### 2.3.5. Adverse Reactions

The occurrence of adverse reactions such as gastrointestinal discomfort, abdominal pain, dry mouth, drowsiness, headache, and other adverse reactions during treatment in the two groups were observed and compared.

#### 2.3.6. Recurrence Rate

All the patients were followed up for two months after treatment. They were informed of the changes of the patients' conditions by phone or return visit every two weeks, and the recurrence situations of the two groups were observed and compared.

### 2.4. Statistical Processing

Data was organized by the double entry method, and SPSS 22.0 statistical software was used for data analysis. The measurement data with normal distribution and homogeneous variance are expressed as (mean ± SD), and the difference between the groups with time factor is analyzed by repeated measures of variance. Differences between the groups without time factor were tested by the independent sample *t*-test, count data were expressed by rate, and the *χ*^2^ test was used. *P* < 0.05 indicated statistical significance.

## 3. Results

### 3.1. Comparison of Clinical Symptom Scores between the Two Groups before and after Treatment

**Figure 1:**
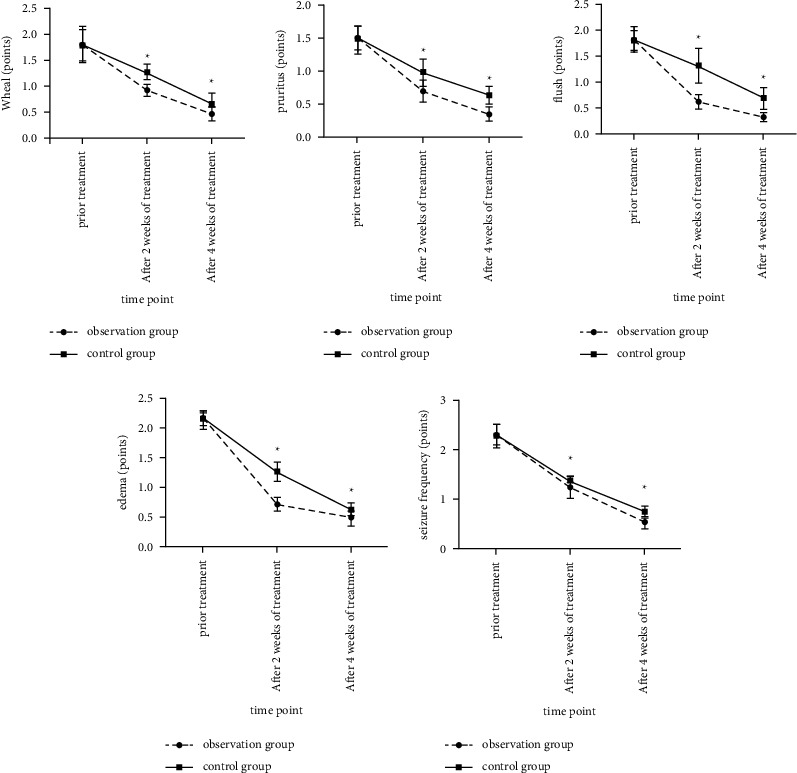
The comparison of clinical symptom scores between the two groups before and after treatment. (Note: compared with the control group, ^*∗*^*P* < 0.05).

The scores of clinical symptoms such as wheal, itching, flushing, edema, and seizure frequency in the observation group at each time point after treatment were lower than those in the control group (*F* time were 725.365, 851.521, 936.411, 3943.136, and 2226.147, all *P* < 0.05; the groups are 40.642, 102.124, 188.523, 259.291, and 23.92, respectively, *P* < 0.05), as shown in Figure 1.

### 3.2. Comparison of Clinical Efficacy between the Two Groups

**Figure 2:**
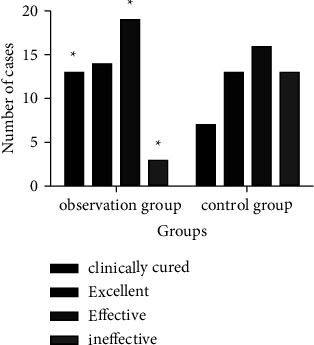
Comparison of clinical efficacy between the two groups. (Note: compared with the control group, ^*∗*^*P* < 0.05.)

The total effective rate of the observation group was 93.88% (46/49), which was significantly higher than 73.47% (36/49) of the control group (*χ*^2^ = 7.470, *P*=0.006), as shown in Figure 2.

### 3.3. Comparison of the Degree of Disease-Related Life Impact between the Two Groups before and after Treatment

**Figure 3:**
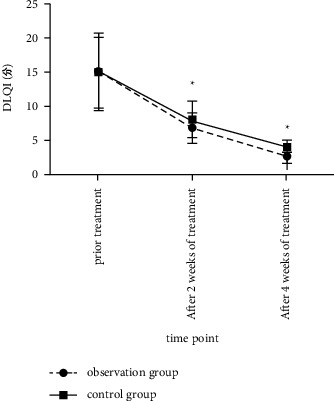
Comparison of the degree of disease-related life impact between the two groups before and after treatment. (Note: compared with the control group, ^*∗*^*P* < 0.05).

The DLQI scores of the observation group at each time point after treatment were lower than those of the control group (*F* time was 282.214, *P* < 0.05; *F* between-group was 6.546, *P* < 0.05), as shown in Figure 3.

### 3.4. Comparison of Serum Complement C3 and C4 Levels between the Two Groups before and after Treatment

**Figure 4:**
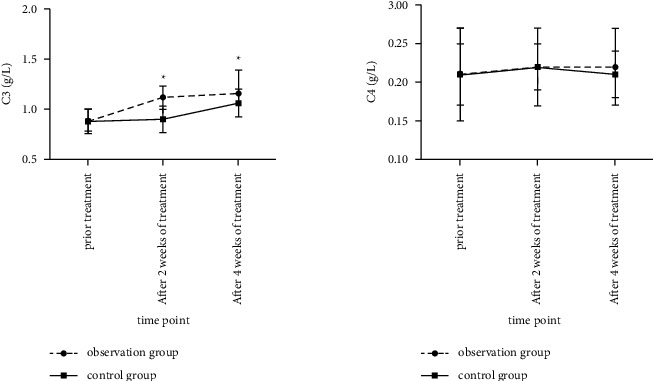
Comparison of serum complement C3 and C4 levels between the two groups before and after treatment. (Note: compared with the control group, ^*∗*^*P* < 0.05).

There was no significant difference in serum C4 levels between the two groups at each time point (*F* time was 1.225, *P* > 0.05; *F* between-group was 0.408, *P* > 0.05); the serum C3 level in the observation group at each time point after treatment was higher than that in the control group (*F* time was 407.352, *P* < 0.05; *F* between-group was 40.951, *P* < 0.05), as shown in Figure 4.

### 3.5. Comparison of Peripheral T Lymphocytes and IgE Levels in the Two Groups before and after Treatment

**Figure 5:**
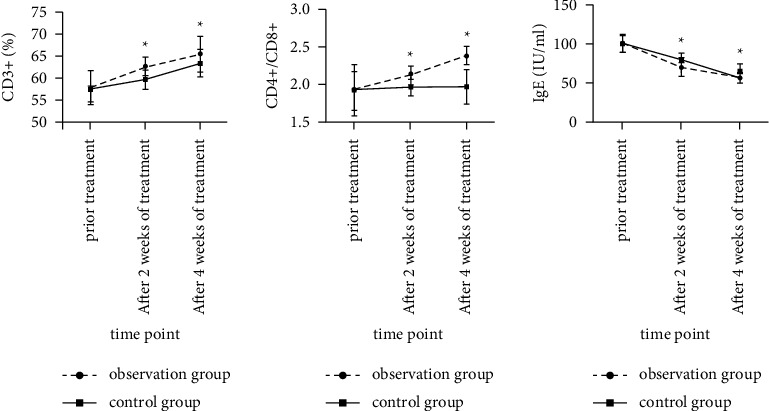
Comparison of peripheral T lymphocytes and IgE levels before and after treatment between the two groups. (Note: compared with the control group, ^*∗*^*P* < 0.05).

The ratio of CD_3_^+^ and CD_4_^+^/CD_8_^+^ in the observation group at each time point after treatment was higher than that in the control group (*F* time were 107.823 and 32.941, *P* < 0.05; *F* between-group were 24.710 and 54.982, *P* < 0.05); the IgE level in the observation group was lower than that in the control group at each time point after treatment (*F* time was 373.124, *P* < 0.05; *F* between-group was 21.802, *P* < 0.05), as shown in Figure 5.

### 3.6. Comparison of IL-4 and IFN-*γ* Levels between the Two Groups before and after Treatment

**Figure 6:**
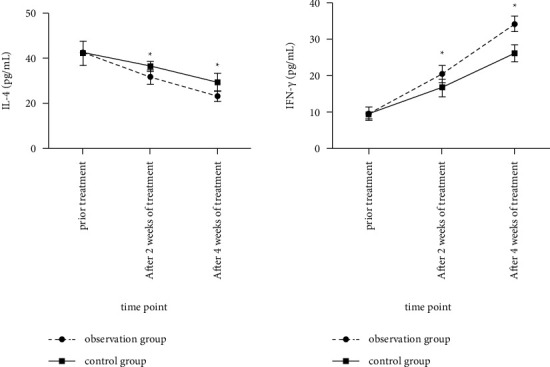
Comparison of the levels of IL-4 and IFN-*γ* between the two groups before and after treatment. (Note: compared with the control group, ^*∗*^*P* < 0.05).

The levels of IL-4 in the observation group were lower than those in the control group at each time point after treatment (*F* time was 395.612, *P* < 0.05; *F* between-group was 62.591, *P* < 0.05); the levels of IFN-*γ* in the observation group were higher than those in the control group at each time point after treatment (*F* time was 2354.147, *P* < 0.05; *F* between-group was 264.921, *P* < 0.05), as shown in Figure 6.

### 3.7. The Incidence of Adverse Reactions and Follow-Up Recurrence in the Two Groups

**Figure 7:**
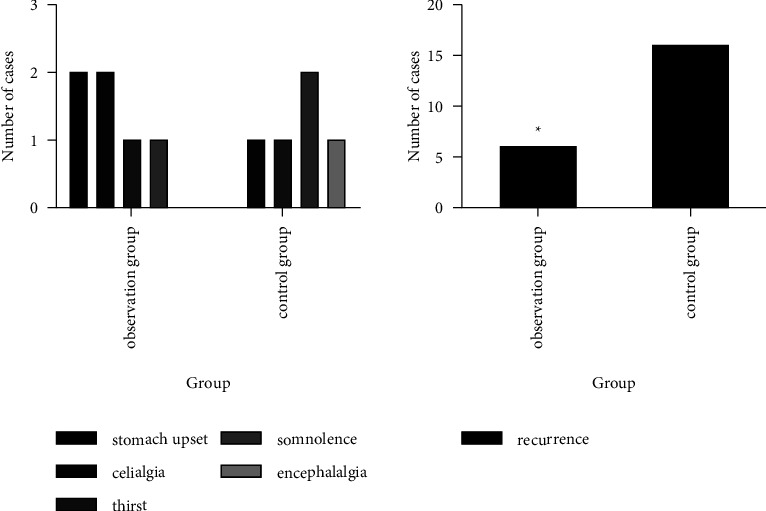
The occurrence of adverse reactions and follow-up recurrence in the two groups. (Note: compared with the control group, ^*∗*^*P* < 0.05).

The incidence of adverse reactions in the observation group was 12.24% (6/49), while that in the control group was 10.20% (5/49), and there was no significant difference in the incidence of adverse reactions between the two groups (*χ*^2^ = 0.102, *P*=0.749). Both the groups were followed up for 2 months after treatment. The recurrence rate was 12.24% (6/49) in the observation group, lower than 32.65% (16/49) in the control group (*χ*^2^ = 5.861, *P*=0.015), as shown in Figure 7.

## 4. Discussion

Chronic urticaria is stubborn and difficult to heal, which brings great inconvenience to the patient's life and has a great adverse impact on the patient's physical, mental, and economic conditions [14]. It is clinically believed that the onset of the disease is closely related to the patient's autoimmunity [15, 16]. Histamine drugs are often used in the treatment of urticaria in modern medicine, which can effectively control the symptoms of skin allergy in patients, but the regulation of the immune system of patients is poor, and it is easy to relapse after drug discontinuation. Therefore, it is necessary to find another drug for synergistic treatment. In China, TCM has been widely used in the treatment of skin diseases in recent years. Clinical efficacy can be effectively improved through TCM syndrome differentiation. It has become a trend to apply TCM therapy to clinical treatment on the basis of modern medicine and so on. In TCM, chronic urticaria is called “addiction rash.” It is more common with gastrointestinal damp-heat syndrome. It is mostly caused by abnormal conduction of the spleen and stomach, resensation of wind pathogen, and damp-heat stagnation on the skin. The main treatment methods are dispelling wind and eliminating dampness, clearing heat and detoxicating. In this study, Fangfeng Tongsheng granule combined with levocetirizine treatment showed that the clinical symptom scores of the observation group at each time point after treatment were lower than those of the control group, and the total clinical effective rate was significantly higher than that of the control group, suggesting that the observation group had better clinical symptoms. The clinical efficacy was better than that of the control group. The results showed that after treatment, the DLQI score of the observation group was significantly lower than that of the control group, which may be related to the significant reduction of the clinical symptoms of the patients. The above results suggest that Fangfeng Tongsheng granule are effective in treating chronic urticaria.

Fangfeng Tongsheng granule are refined from Fangfeng, Nepeta spike, mint, ephedra, rhubarb, Glauber's salt, gardenia, talc, platycodon, gypsum, Chuanxiong, Angelica, white peony, skullcap, forsythia, licorice, and fried Atractylodes. Among them, Fangfeng has the effects of dispelling wind and relieving external appearance, removing dampness and activating collaterals and regulating the spleen and stomach; Nepeta paniculata has the effect of sweating, expelling wind, and relieving pain; peppermint has the effect of dispersing wind-heat, detoxifying, and expelling rash; Ephedra has the function of diaphoresis, diuresis, and detumescence; Rhubarb has the effects in relieving constipation, clearing heat, relieving fire, and detoxicating; Glauber's salt has the effect of eliminating fire and swelling, clearing away heat, and detoxifying; gardenia has the effect of relieving heat and dampness, dispelling fire, and eliminating vexation; talc has the effects of clearing away heat and toxic materials, eliminating dampness, and astringing sore; *Platycodon grandiflorum* has the effect of nourishing qi and strengthening the spleen; gypsum has the effects of clearing heat, purging fire, and promoting tissue regeneration to relieve pain; Chuanxiong has the effects of promoting blood circulation and promoting Qi, drying dampness, and dispelling wind; white peony has the effects of reconciling nutrition, nourishing blood, and relieving pain; Scutellaria baicalensis has the effects of clearing away heat and dampness, purging fire, and detoxifying; forsythia has the effects of dispelling wind and dissipating heat, clearing heat, and detoxifying; licorice has the effects of invigorating the spleen and qi, and reconciling medicinal properties; fried Atractylodes has the effects of drying dampness and water, nourishing qi and invigorating the spleen. The whole formula is multidrug compatibility, taking into account both the exterior and the interior, focusing on expelling wind, followed by clearing heat, diuresis, and dehumidification, nourishing blood and qi, strengthening the spleen and nourishing the stomach, supplemented by the functions of dispelling wind and detoxifying, clearing heat and detoxifying, and promoting blood circulation and nourishing qi, and combined with levocetirizine can effectively improve the clinical symptoms of patients.

Earlier, we mentioned that the immune system is related to the pathogenesis of chronic urticaria. Serum complement plays an important role in the body's immune system. Complement C3 is the most abundant complement component in serum. It is involved in cellular immunity, can recognize and remove foreign pathogens, and can activate the part of complement protein, kills invading bacteria [17–19]. CD_3_^+^, CD_4_^+^, and CD_8_^+^ are all cellular immune regulatory T cell subsets. In addition, the ratio of CD_4_^+^ to CD_8_^+^ is a sensitive indicator of human immunodeficiency, and the decrease of this ratio indicates that the body's immune function is inhibited [20–22]. IgE is closely related to the pathogenesis of chronic urticaria and is the key immunoglobulin that mediates the pathogenesis of the disease [23]. CD_4_^+^ lymphocytes can differentiate into Th1, Th2, and other subgroups under the stimulation of antigens, regulate cellular immunity, and Th2 cells secrete IL cytokines such as −4, stimulate B cells to switch antibody classes, produce IgE antibodies, and IgE binds to Fc receptors in basophils and mast cells to activate allergic reactions. IFN-*γ* is secreted by Th1 cells, which can promote the differentiation of T0 cells to T1 cells and inhibit the differentiation of T0 cells to T2 cells [24–26]. The results showed that after treatment, the serum C3 complement, IFN-*γ* levels and CD_3_^+^, CD_4_^+^/CD_8_^+^ ratios in the observation group were higher than those in the control group, while the levels of IgE and IL-4 were lower than those in the control group. It shows that the combined treatment of Fangfeng Tongsheng granule can effectively improve the immune status of patients, but its specific mechanism still needs to be further studied. Chuanxiong, Angelica, Atractylodes, and other medicines in Fangfeng Tongsheng granule also have the effect of enhancing human immunity. The study showed that there was no significant difference in the incidence of adverse reactions between the two groups of patients, suggesting that the addition of Fangfeng Tongsheng granule may not increase the risk of adverse reactions. All patients were followed up for two months and the recurrence rate in the observation group was lower than that in the control group. This indicated that the combination of Fangfeng Tongsheng granule and levocetirizine could effectively reduce the recurrence risk of urticaria in patients after drug discontinuation, which might be related to the improvement of immune status in patients with Fangfeng Tongsheng granules.

In conclusion, the application of Fangfeng Tongsheng granules combined with levocetirizine in patients with chronic urticaria can effectively improve the clinical symptoms, improve the clinical efficacy, reduce the impact of the disease on life, improve the immune status of patients, and reduce the risk of recurrence with better efficacy and safety.

## Data Availability

The data can be obtained from the corresponding author upon reasonable request.
